# Disruption of Spectrin-Like Cytoskeleton in Differentiating Keratinocytes by PKCδ Activation Is Associated with Phosphorylated Adducin

**DOI:** 10.1371/journal.pone.0028267

**Published:** 2011-12-07

**Authors:** Kong-Nan Zhao, Paul P. Masci, Martin F. Lavin

**Affiliations:** 1 University of Queensland Centre for Clinical Research, The University of Queensland, Brisbane, Queensland, Australia; 2 Centre for Integrative Clinical and Molecular Medicine, School of Medicine, The University of Queensland, Princess Alexandra Hospital, Woolloongabba, Brisbane, Queensland, Australia; 3 Queensland Institute of Medical Research, Herston, Brisbane, Queensland, Australia; University of Birmingham, United Kingdom

## Abstract

Spectrin is a central component of the cytoskeletal protein network in a variety of erythroid and non-erythroid cells. In keratinocytes, this protein has been shown to be pericytoplasmic and plasma membrane associated, but its characteristics and function have not been established in these cells. Here we demonstrate that spectrin increases dramatically in amount and is assembled into the cytoskeleton during differentiation in mouse and human keratinocytes. The spectrin-like cytoskeleton was predominantly organized in the granular and cornified layers of the epidermis and disrupted by actin filament inhibitors, but not by anti-mitotic drugs. When the cytoskeleton was disrupted PKCδ was activated by phosphorylation on Thr505. Specific inhibition of PKCδ^(Thr505)^ activation with rottlerin prevented disruption of the spectrin-like cytoskeleton and the associated morphological changes that accompany differentiation. Rottlerin also inhibited specific phosphorylation of the PKCδ substrate adducin, a cytoskeletal protein. Furthermore, knock-down of endogenous adducin affected not only expression of adducin, but also spectrin and PKCδ, and severely disrupted organization of the spectrin-like cytoskeleton and cytoskeletal distribution of both adducin and PKCδ. These results demonstrate that organization of a spectrin-like cytoskeleton is associated with keratinocytes differentiation, and disruption of this cytoskeleton is mediated by either PKCδ^(Thr505)^ phosphorylation associated with phosphorylated adducin or due to reduction of endogenous adducin, which normally connects and stabilizes the spectrin-actin complex.

## Introduction

Spectrin is a high molecular weight protein, with a wide range of physiological functions [1], [2]. This protein comprises α (240 kDa) and β (220 kDa) subunits, which associate to form an elongated (αβ)2 tetramer [1]. Located close to the interior surface of the plasma membrane, spectrin forms a hexagonal lattice, the nodes of which are cross-linked by the cytoskeletal protein actin [1]. Spectrin was first identified as a central component of the cytoskeletal protein network [3], and was thought to be erythrocyte-specific [4], [5]. The identification of a non-erythroid spectrin-like protein [6] was followed by the widespread discovery of avian spectrin in non-erythroid cells [7], [8]. Spectrin plays a wide variety of functions including axonal transport, neurite extension and protein sorting in the Golgi apparatus and cell membrane in erythrocytes. This protein also enables red blood cells to pass through capillaries, confers elasticity on the cell, maintains the discoid shape of the membrane and organization of synaptic vesicles and restricts the lateral mobility of its macromolecules [9]. However, whether spectrin has the same functions in non-erythrocytes remained unknown over two decades [1]. Further studies in *C. elegans* proved that β -spectrin is required for a subset of processes at cell membranes [10], [11]. The loss of β -spectrin leads to abnormal axon outgrowth in neurons, to disorganization of the myofilament lattice, discontinuities in the dense bodies, and a reduction or loss of the sarcoplasmic reticulum in muscles [10], [11]. Genetic analysis in *Drosophila* provides evidence that β-spectrin mutations are lethal during late embryonic/early larval development and this protein plays a role in determining the subcellular distribution of the Na^+^, K^+^ ATPase [12].

Keratinocytes are the major cell type in the epidermis responsible for constructing the protective barrier of mammalian skin by undergoing a complex and carefully choreographed program of cell differentiation [13]. Proliferative keratinocytes in the basal layer periodically detach from an underlying basement membrane of extracellular matrix and move outward. Once in the suprabasal layer, keratinocytes stop dividing and enter a differentiation program. Terminally differentiated keratinocytes flatten and develop the cornified envelopes, which consist of a stabilized array of keratin filaments contained within a covalently cross-linked protein envelope and play a critical protection role in barrier function of the epithelium [14]. Without this protection, the epithelium would quickly hydrate in wet environments, dehydrate in arid environment and be extremely susceptible to infection by pathogens [15], [16]. The best evidence of the importance of this barrier for survival is the heroic efforts required to assist burn victims in regulating fluid balance and remaining free of infection [15]. In keratinocytes, non-erythrocyte spectrin is pericytoplasmic and plasma membrane-associated [17], [18]. However, to date, the characteristics and function of the spectrin-like cytoskeleton in keratinocytes are not well understood. Here, we studied spectrin protein expression and organization of the spectrin-like cytoskeleton in both mouse and human keratinocytes *in vitro* and *in vivo*. We showed that this membrane-bound protein expression and its cytoskeletal organization were associated with keratinocyte differentiation. We also showed that the spectrin-like cytoskeleton in differentiating keratinocytes *in vitro* was disrupted by actin microfilament inhibitors and by β-adducin siRNA. We demonstrated that disruption of the spectrin-like cytoskeleton associated with keratinocyte differentiation and cellular integrity was mediated through PKCδ activation associated with phosphorylation of adducin or expression of endogenous adducin.

## Methods

### Antibodies

Primary antibodies (Abs) used were rabbit polyclonal Abs to human spectrin (S1515) and to actin; mouse monoclonal Abs to β-tubulin and to protein kinase C (PKC). All were from Sigma-Aldrich (Australia). Rabbit polyclonal antibodies to involucrin and K14 were purchased from Covance (USA). Mouse monoclonal Ab to αI spectrin, rabbit polyclonal antibodies to PKCδ, to adducin β and p-adducin^(Ser662)^ and goat polyclonal antibodies to βI spectrin and p-PKCδ^(Thr505)^ were purchased from St Cruz (USA). Rabbit polyclonal antibodies to PKCα, p-PKCα^(Ser 657)^ and p- PKCα^(Thr638/641)^ were purchased from Cell Signalling (USA).

### Primary keratinocyte culture

Primary keratinocytes were isolated from new born mouse skin as previously described [19]. Isolated keratinocytes were grown as adherent cultures in a freshly prepared medium (365 ml DMEM medium, 2 mM glutamine, 100 unit/ml penicillin, 100 unit/ml streptomycin, 125 ml Hams F12 medium, 50 ml FBS, 2.5 mg transferrin, 2.5 mg insulin, 4.2 µg cholera toxin, 0.12 mg hydrocortisone, 17 mg adenine and 10 mg gentamicin) for one day (D1) and then cultured in KC-SFM medium with low concentration of calcium (0.2 mM) (GIBCO, Australia) for three (D4) and six (D7) days. This low concentration of calcium could induce the keratinocytes commitment to terminal differentiation within a week based on our previous studies [19], [20]. The cultured keratinocytes at three time points (D1, D4 and D7) were collected for examining spectrin expression and organization of spectrin-like cytoskeleton.

### Inhibitor treatments

**(A).** Treatment with anti-mitotic drugs and microfilament inhibitors. Primary keratinocytes grown *in vitro* for five days were treated with two anti-mitotic drugs (3 µM nocodazole (Noc) and 3 µM colchicines (Col)) and three microfilament inhibitors (4 µM cytochalasin B (CB), 1.2 µM staurosporine (STS) and 1.2 µM latrunculin B [21]) for 12 h. **(B).** Co-treatment with rottlerin (RT) and microfilament inhibitors. Primary keratinocytes grown *in vitro* for five days were treated either with RT (2 µM) and three microfilament inhibitors (4 µM CB, 1.2 µM STS and 1.2 µM Lat) alone; or co-treated with RT and one of three microfilament inhibitors respectively for 12 h. The inhibitor-treated keratinocytes were fixed for immunofluorescence microscopy and collected for protein preparation for Western blotting.

### β-Adducin siRNA transfection

One control siRNA(SC-37007; St Cruz, USA) and one β-adducin siRNA that consist of pools of three to five target-specific 19–25 nt siRNAs designed to knockdown gene expression (SC-37061, St Cruz, USA) were used for knock-down of endogenous adducin expression in primary mouse keratinocytes. The primary mouse keratinocyte culture at D5 with 80% confluence were transfected with the two mouse siRNAs using siRNA transfection reagents (St Cruz, USA) following the manufacturer's directions. siRNA transfected-keratinocytes were either harvested for preparation of protein samples, or fixed for immunofluorescence labeling at 48 h post- transfection.

### Immunofluorescence labelling

Keratinocytes cultured for one, four and seven days were fixed and permeabilized with 85% ethanol for 10 min. Fixed keratinocytes were blocked with 5% skim milk-PBST and probed with a polyclonal antibody against αβ-spectrin (Sigma) followed by a fluorescein-isothiocyanate-conjugated (FITC) secondary antibody (Sigma). The spectrin labelled keratinocytes were further probed with Cy3-conjugated monoclonal antibody against tubulin (Sigma) or with a polyclonal antibody against involucrin (Covance, USA) and followed by a Cy3-conjugated secondary antibody (Sigma). Nuclei were counterstained with 4′, 6′-diamidino-2-phenylindole (DAPI). Immunofluorescence labelling was similarly performed on paraffin sections of mouse skin and human foreskin using a polyclonal antibody against αβ-spectrin (Sigma, Australia) and followed by a FITC secondary antibody. The spectrin labelled skin sections were further blocked with 5% skim milk-PBS and probed with Cy3-conjugated monoclonal antibody against tubulin (Sigma), or with a polyclonal antibody against keratin K14 (Covance, USA), and or with a polyclonal antibody against involucrin (Covance, USA) and followed by Cy3-conjugated secondary antibody (Sigma). Nuclei were counterstained with DAPI. Fluorescent staining was photographed using a Zess Eclipse 800 microscope equipped with a DXM1200 digital camera.

### Western blot analysis

Cultured primary mouse and human keratinocytes were collected for protein preparation. Cell pellets were lysed in lysis buffer, pH 7.4, containing 2 mM phenylmethylsulfonyl fluoride (PMSF), 2 µg/ml of aprotinin; 2 µg/ml of benzamidine (Sigma) and 1 µg/ml of leupeptin (Auspep, Australia) and sonicated for 40 sec. Forty µg protein samples were separated by 6% polyacrylamide gel and blotted onto PVDF membrane. The blots were first probed with either polyclonal antibodies against αβ-spectrin, or involucrin, or PKCδ, P-PKCδ, or adducin and P-adducin, or actin, and or a monoclonal antibody against β-tubulin. The blots were then probed with horseradish-peroxidase-conjugated goat anti-rabbit or mouse IgG (Sigma) and visualized using a chemiluminescence system. Alternatively, the blots were first probed with the polyclonal antibodies against either αβ-spectrin or α- and β-spectrin in Odssey blocking buffer. The blots were then probed with secondary donkey anti-rabbit antibody (IRDye®680, Odyssey) and visualized using an Odyssey infrared imaging system.

### Immunoprecipitation

Keratinocytes, after washing with phosphate-buffered saline, lysing in 1 ml of lysis buffer (50 mM Tris-HCl, pH 8, containing 150 mM NaCl, 1% NP-40, 1 mM MgCl2, 1 mM EDTA, 2 mM PMSF, 2 µg/ml leupeptin, 2 µg/ml aprotinin and 1 µg/ml pepstatin) were sonicated and centrifuged at 12,000 *g* at 4°C for 5 min. The supernatant (0.5 ml) was precleared with 50 µl of protein G beads, previously washed with 500 µl of lysis buffer for 1 h at 4°C. Antibodies either monoclonal or polyclonal against different target proteins were added to the precleared supernatant, respectively, with 1.5 µg of antibody to 400 µg of protein. The protein sample with antibody was incubated for 2 h at 4°C with gentle agitation. The complex was precipitated by incubating with 20 µl of protein-A/G PLUS agarose (Santa Cruz Biotechnology) over night at 4°C. The mixture was then centrifuged, and the pellet was washed 5 times with lysis buffer. The pellet was resuspended in 1× Laemmli buffer, boiled, electrophoresesed on a 7.5% polyacrylamide gel and transferred to PVDF membrane for immunobloting assay.

### Animal and human research ethic statement

Animal research ethics approval for this study using primary keratinocytes isolated from new born mouse skin for *in vitro* culture and adult mouse skin materials was obtained from The University of Queensland Animal Ethics Committee (AEC No: CICR/196/06/CICR). Human research ethic approval for this study using human skin materials was obtained from Medical Research Ethics committee of The University of Queensland (MEC No: MED/PAH/04/NHMRC/UNIQUE). The primary human keratinocytes used for supplementary experiments in this study were kindly provided by Associate Professor Nicholas A. Saunders at the Diamantina Institute for Cancer Immunology and Metabolic Medicine, The University of Queensland. Dr. Saunders had the consent from the donors. Therefore, The Medical Research Ethics committee of The University of Queensland waived the need for consent from the donors for using the human foreskin to prepare primary human keratinocytes.

## Results

### A spectrin-like cytoskeleton is organized in differentiating keratinocytes *in vitro*

**Figure 1:**
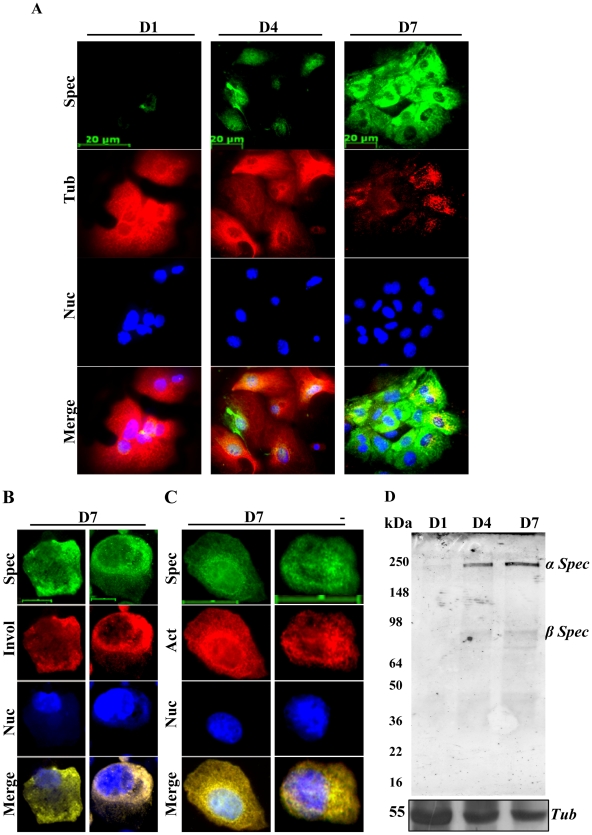
Spectrin-like cytoskeleton and spectrin expression in primary mouse keratinocyte cultures up to seven days. (**A**). Keratinocytes cultured for 1 (**D1**), 4 (**D4**) and 7 (**D7**) days were compared for spectrin-like cytoskeleton (Green) and tubulin cytoskeleton (Red) by immunofluorescence staining. (**B**). Immunofluorescence staining for spectrin (Green) and involucrin a terminal differentiation marker (Red) in **D7** cultured keratinocytes. (**C**). Immunofluorescence staining for spectrin (Green) and actin (Red) in **D7** cultured keratinocytes. Nuclei (Blue) from the same field were counterstained with 4′,6′-diamidino -2-phenylindole (DAPI). Scale bars are 15 µm in (**A**) and in (**B**) and 20 µm in (**C**). (**D**). Western blot analysis for spectrin and tubulin in primary keratinocytes cultured for a period of 1–7 days. Results are representative of three individual experiments.

We previously confirmed progressive differentiation of primary mouse keratinocytes cultured *in vitro* in low calcium medium at a specific cell density from day 1 to day 7 [19], [22]. Here, we first examined the spectrin-like cytoskeleton and spectrin expression in primary mouse and human keratinocyte cultures over a period of seven days. The majority of primary mouse and human keratinocytes grown *in vitro* at D1 failed to show spectrin staining (Fig. 1A; Supporting information Fig. S1), but punctate spectrin staining was evident in a few cells (Fig. 1A; Supporting information Fig. S1), indicating weak expression of spectrin protein that appeared to be intracytoplasmic. By D4, 30–50% of keratinocytes showed staining of spectrin, which was associated with the nuclei (Fig. 1A). By D7, 60–80% of the cultured keratinocytes stained strongly for spectrin and had a well organized spectrin-like cytoskeleton across the plasma membrane (Fig. 1A; Supporting information Fig. S1). In D7 keratinocytes, spectrin was co-expressed with the keratinocyte differentiation marker involucrin (Fig. 1B). Immunofluorescence staining showed a co-localization of spectrin and actin in D7 keratinocytes (Fig. 1C). Immunoblotting confirmed further that expression of spectrin in both primary mouse and human keratinocyte cultures was significantly up-regulated with increasing culture time, coincident with increased expression of involucrin (Supporting information Fig. S2). The antibody used was raised to detect both α- and β- spectrins (240 and 220 kDa), but Immunoblotting showed only one spectrin band of 240 kDa (Fig. 1D). Using antibodies specific to α1 spectrin and β1 spectrin, it was confirmed that the spectrin band detected corresponds to α spectrin at 240 kDa (Data not shown). Furthermore, β1 specific spectrin antibody confirmed that the faint band observed at approximately 78 kDa (Fig. 1D), probably corresponded to an alternative form of β spectrin (SPTBN4 with 678 amino acids; http://biogps.gnf.org). All of the D1 cultured keratinocytes showed strong tubulin staining and a well organized tubulin cytoskeleton (Fig. 1A; Supplementary Figure S2B), but by D7, tubulin staining was reduced and the tubulin cytoskeleton was not well organized (Fig. 1A; Supporting information Fig. S1). Expression of tubulin was down-regulated (Fig. 1D; Supplementary Figure S2A and S3B). This is consistent with previous data showing that undifferentiated keratinocytes contained numerous microtubules radiating from a centrosomal organization center (MTOC) [23], while differentiating keratinocytes displayed an altered cytoskeleton including rearranged microtubules [23], [24]. These results suggest that spectrin and tubulin are inversely related in their expression and organization in cytoskeletal structures during keratinocyte differentiation.

### Spectrin-like cytoskeleton is predominantly organized in granular and cornified layers of the epidermis

**Figure 2:**
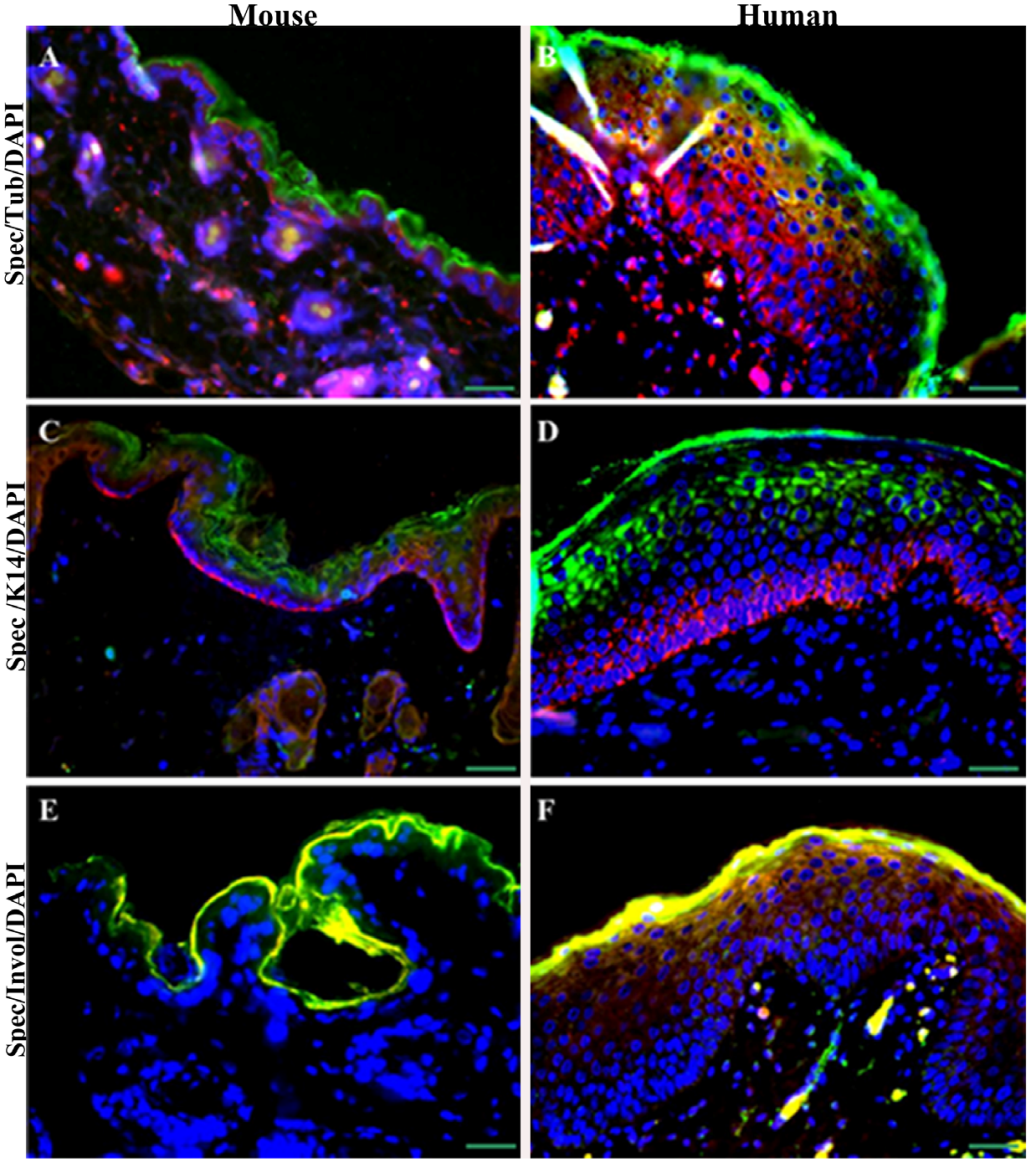
Organization of spectrin-like cytoskeleton, microtubules, K14 and involucrin filaments in mouse and human skin. (**A and B**). Sections of mouse and human skin were immunostained as indicated for spectrin(Green) and tubulin (Red). (**C and D**). Sections of mouse and human skin were immunostained as indicated for spectrin (Green) and K14 (Red). (**E and F**). Sections of mouse and human skin were immunostained as indicated for spectrin (Green) and involucrin (Red). Nuclei (Blue) from the same fields were counterstained with DAPI. Scale bars are 30 µm. The mouse has a narrow staining skin epidermis while that in human shows greater depth of staining.

To investigate this phenomenon in greater depth, we examined the spectrin-like and microtubule cytoskeletons in both mouse and human skin using immunofluorescence microscopy (Fig. 2A–F; Supporting information Fig. S3, S4, S5). Strong staining of spectrin protein was observed in granular keratinocytes and cornified envelope where nuclei are being lost [25]. This is different from a previous observation that spectrin-like proteins are localized to the peripheral cytoplasm of basal keratinocytes [26]. Spectrin staining was also observed in papillae of mouse dermis and in superficial differentiated keratinocytes in deeper hair follicles of human epidermis (Fig. 2A–F; Supporting information Fig. S3). In contrast, tubulin stained more extensively throughout the dermis and epidermis including the basal, suprabasal and granular keratinocytes, except for the cornified envelope (Fig. 2A–B; Supporting information Fig. S3). No staining of the spectrin-like cytoskeleton was observed in basal keratinocytes of the proliferative zone, which showed typical staining of basal cell keratin K14 (Fig. 2C–D; Supporting information Fig. S4), consistent with previous data showing that K14 is expressed exclusively in the innermost mitotically active basal cell layer in normal skin [27]. Staining patterns of spectrin-like cytoskeleton in mice and human skin were closely associated with involucrin (Fig. 2E–F; Supporting information Fig. S5). Our data reveal a characteristic feature of the spectrin-like cytoskeleton predominantly organized in granular and cornified layers of the epidermis, which is associated with stratified epithelia in mouse and human skin.

### Two microfilament inhibitors disrupt the spectrin-like cytoskeleton in keratinocytes

**Figure 3:**
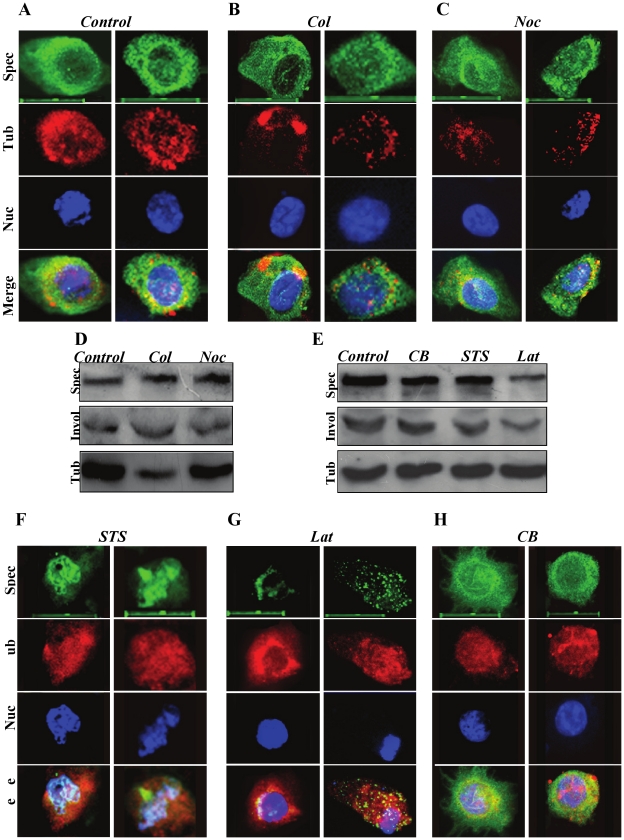
Disruption of the spectrin-like cytoskeleton in primary mouse keratinocytes by cell division and microfilament inhibitors. **A**), **B**) and **C**). Keratinocytes treated with cell division inhibitors were compared for organization of spectrin cytoskeleton (Green) and tubulin (Red) in primary keratinocytes by immune-fluorescence staining. Nuclei (Blue) from the same field were counterstained with DAPI. Scale bars are 20 µm. **D**) and **E**). Western blot analysis for spectrin, involucrin and tubulin in primary mouse keratinocytes treated with cell division and microfilament inhibitors. **F**), **G**) and **H**). Keratinocytes treated with microfilament inhibitors were compared for organization of spectrin cytoskeleton (Green) and tubulin (Red) by immune-fluorescence staining. Nuclei (Blue) from the same field were counterstained with DAPI. Scale bars are 20 µm.

To investigate the possible mechanism that may regulate assembly of the spectrin-like cytoskeleton associated with keratinocyte differentiation, we used different cytoskeletal inhibitors to treat the primary mouse keratinocytes in culture. We first treated the primary mouse keratinocytes, grown in medium for five days, with two anti-mitotic drugs: colchicine (Col) and nocodazole (Noc) for 12 h to investigate the possible mechanism that regulates organization of the spectrin-like cytoskeleton during keratinocyte differentiation. These two drugs had little effect on the spectrin-like cytoskeleton in keratinocytes (Fig. 3A–C). Western blot analysis confirmed that neither Col nor Noc altered spectrin expression (Fig. 3D). Only Col markedly reduced tubulin expression (Fig. 3D). The results suggest that polymerization and assembly of the spectrin-like cytoskeleton and spectrin expression associated with keratinocyte differentiation are independent of cell division because keratinocytes stop dividing after entering a differentiation program [14], [28].

We next examined whether actin filament inhibitors could affect organization of the spectrin-like cytoskeleton and protein expression in keratinocytes. Three actin filament inhibitors: Cytochalasin B (CB), staurosporine (STS) and latrunculin B [21] were employed. Actin filaments in keratinocytes treated with the three inhibitors were severely disrupted (Supporting information Fig. S6). Both STS and Lat treatments of keratinocytes not only disrupted actin filaments, but also dissolved the spectrin-like cytoskeleton over the cell membrane, resulting in curved and short spectrin filaments centralized on the nuclear surface (Fig. 3F–H). However, CB treatment did not affect organization of the spectrin-like cytoskeleton (Fig. 3H). In contrast, immunofluorescence staining for tubulin cytoskeleton was clearly stronger in all three actin inhibitor-treated keratinocytes (Figure 3F–H) than in untreated cells (Figure 3A). Western blots showed that the levels of spectrin protein were slightly reduced in keratinocytes treated with CB and STS, but markedly reduced in Lat-treated keratinocytes (Fig. 3E). The levels of tubulin in keratinocytes treated with the actin polymerization inhibitors were not affected (Fig. 3E). Immunoblotting did not show any evidence of spectrin degradation (Supporting information Fig. S7). These data demonstrate that both STS and Lat treatments caused qualitative and quantitative changes of the spectrin-like cytoskeleton, which can explain abnormalities of cell shape in keratinocytes, similar to previous studies in erythrocytes [29], [30].

### Disruption of the spectrin-like cytoskeleton by actin filament inhibitors is accompanied by phosphorylation of PKCδ^(Thr505)^

**Figure 4:**
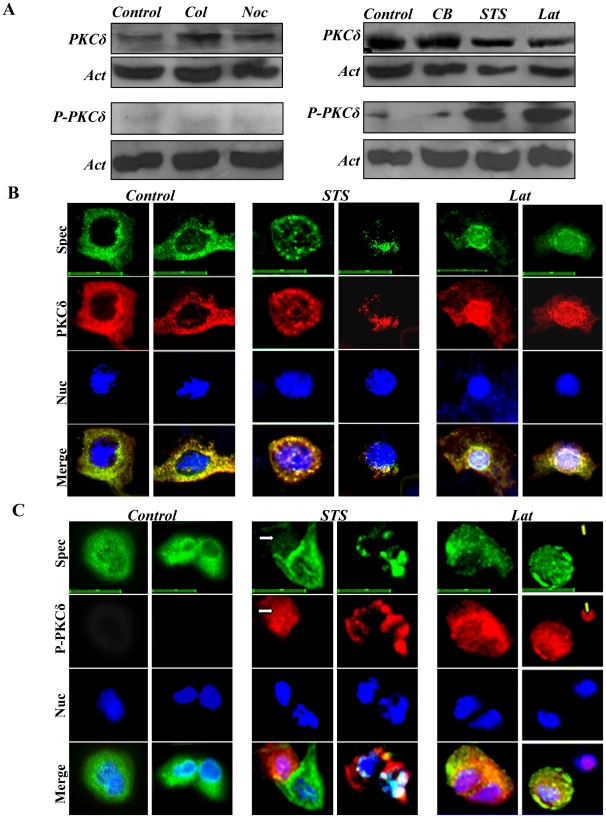
Differential effects of cell division and microfilament inhibitors on expression of PKCδ and phosphorylated-PKCδ^(Thr505)^ (labelled as P-PKCδ) in primary keratinocytes. Primary mouse keratinocytes, after culturing for five days, were treated with the five inhibitors for 12 h, respectively. (**A**) Western blot analysis for expression of PKCδ (upper panel) and P-PKCδ (lower panel)after treatment with different inhibitors. Actin was examined as loading control. (**B**). Control and STS- and Lat-treated keratinocytes were triple-stained for spectrin (Green), PKCδ (Red) and nuclei (Blue). Scale bars are 15 µm. (**C**). Control and STS- and Lat-treated keratinocytes were triple-stained for spectrin (Green), P-PKCδ (Red) and nuclei (Blue). Arrows point to cells exhibiting strong phosphorylation of PKCδ^(Thr505)^ and weak expression of spectrin. Scale bars are 20 µm.

**Figure 5:**
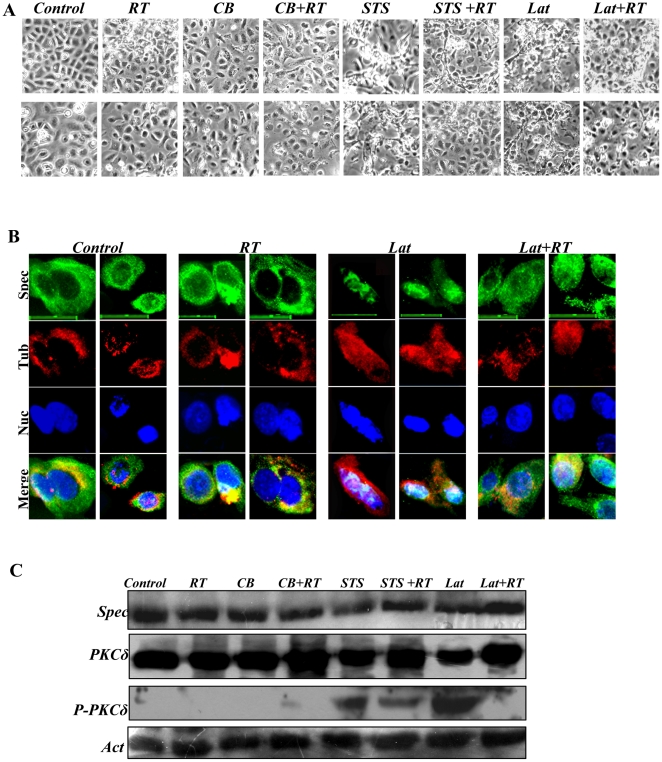
Rottlerin (RT) inhibited expression of P-PKCδ caused by STS and Lat in primary mouse keratinocytes. Primary mouse keratinocytes after culturing for five days were treated with RT, CB, CB+RT, STS, STS+RT, Lat and Lat+RT for 12 h, respectively. The keratinocytes were then recorded for their morphology and harvested for analysis of expression of spectrin, PKCδ and P-PKCδ. 2 µm RT was used. (**A**). Cell morphology comparison between control and inhibitor-treated keratinocytes. (**B**). Cells were compared for organization of spectrin-like cytoskeleton (Green) and tubulin cytoskeleton (Red) in primary mouse keratinocytes treated with control, RT, Lat and Lat+RT by immunofluorescence staining. Scale bars are 20 µm. (**C**). Western blotting analysis for expression of spectrin, PKCδ and P-PKCδ. Actin was examined as loading control.

Keratinocytes express the five major isoforms of protein kinase C (PKC) [31], but PKCα and PKCδ are the most abundant isoforms, regulating proliferation and differentiation of these cells [32], [33], [34], [35], [36]. Thus, we determined whether the three actin filament inhibitors affected expression of PKCα and PKCδ in keratinocytes. Immunoblotting analysis failed to reveal any effect of the three inhibitors on expression of PKCα in keratinocytes (Supporting information Fig. S8). Furthermore, phosphorylation of PKCα on Ser657, or Thr638/641 was not detected in STS- and Lat-treated keratinocytes (data not shown). The two anti-mitotic drugs (Col and Noc) had little or no effect on expression of PKCδ (Fig. 4A, left hand panel). Expression of PKCδ appeared to be reduced in both STS and Lat–treated keratinocytes, compared with that in control and CB-treated keratinocytes (Fig. 5A, right hand panel). Both STS and LAT markedly enhanced phosphorylation of this protein on Thr505 (P-PKCδ^(Thr505)^; Fig. 4A, right hand panel), but CB had no effect. Phosphorylation at this site increases the activity of PKCδ by up to 80-fold [37]. The mitotic inhibitors (Col and Noc) did not alter expression of P-PKCδ^(Thr505)^ above basal levels (Fig. 4A, left hand panel). Immunofluorescence staining revealed a PKCδ cytoskeleton distribution in untreated keratinocytes, similar to that of the spectrin-like cytoskeleton (Fig. 4B). Treatment with STS and Lat resulted in dissolution of the spectrin-like cytoskeleton and abnormal re-organization of both spectrin and PKCδ in the cytoplasm and nucleus (Fig. 4*B*). It has been reported that both spectrin and PKCδ bind actin and predominantly localize to the actin filaments [38], [39]. Latrunculin decreases PKCδ binding to actin by directly binding to it and thereby preventing its polymerization [38], [40]. Consistent with the increased phosphorylation of PKCδ observed by immunoblotting after STS and LAT treatments, intense staining for P-PKCδ^(Thr505)^ was observed in keratinocytes treated with these agents (Fig. 4C). In addition, spectrin staining was distinctly diminished under these conditions (Fig. 4C). The results indicate that phosphorylation of PKCδ^(Thr505)^ is tightly associated with disruption of the spectrin-like cytoskeleton in keratinocytes.

### Inhibition of PKCδ activation by rottlerin prevents disruption of the spectrin cytoskeleton

We determined further whether phosphorylation of PKCδ on Thr505 disrupts the spectrin-like cytoskeleton and inhibits expression of spectrin protein, using rottlerin (RT), a specific inhibitor of PKCδ phosphorylation in different mammalian cells [41]. Co-treatment with STS and RT or Lat and RT provided a reversal of the morphological changes of the cells (Fig. 5A), and also prevented disruption of the spectrin-like cytoskeleton associated with keratinocytes differentiation (Fig. 5B). Concomitant with this RT also prevented the decrease in spectrin (Fig. 5C). Under these conditions RT reduced PKCδ phosphorylation in both STS and especially Lat treated cultures (Fig. 5C). Furthermore, co-treatments of STS and RT or Lat and RT scarcely affected expression of pan-PKC and PKCα (Data not shown). The results support further an important role for phosphorylation of PKCδ^(Thr505)^, inhibiting spectrin expression and disrupting the organization of the spectrin-like cytoskeleton in differentiating keratinocytes.

### Phosphorylation of PKCδ appears not to directly disrupt organization of spectrin-like cytoskeleton

**Figure 6:**
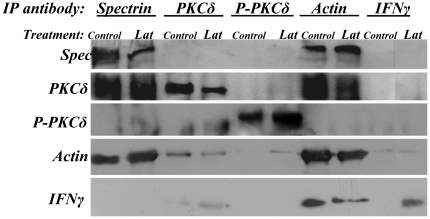
Immunoprecipitation assay of total proteins prepared from primary mouse keratinocyte cultures with the indicated antibodies. Primary mouse keratinocytes cultured for five days were treated with or without Lat for 12 h. The keratinocytes were then harvested for preparing protein samples. Protein samples were immunoprecipitated with the five indicated antibodies and analyzed by immunoblotting assay.

We determined whether P-PKCδ^(Thr505)^ interacted with spectrin protein to directly disrupt the spectrin-like cytoskeleton in differentiating keratinocytes. Immunoprecipitation with antibodies against involucrin, tubulin, pan-PKC and PKCα did not precipitate spectrin (Supporting information Fig. S9), indicating that the four proteins examined do not interact with spectrin, suggesting further that they are not directly involved in re-organization of the spectrin-like cytoskeleton. Anti-actin antibody precipitated spectrin, but neither anti-PKCδ, P-PKCδ^(Thr505)^, nor IF-γ (as a negative control) antibody precipitated spectrin (Fig. 6). Both anti-spectrin and actin antibodies, but not P-PKCδ^(Thr505)^ and IF-γ antibodies, precipitated PKCδ in cell lysates (Fig. 6). None of the four antibodies could precipitate P-PKC^(Thr505)^ (Fig. 6). Thus, it appears that activation of PKCδ on Thr505 does not directly disrupt organization of the spectrin-like cytoskeleton.

### Adducin, a potential substrate for PKCδ and P-PKCδ^(Thr505)^ in disruption of the spectrin-like cytoskeleton

**Figure 7:**
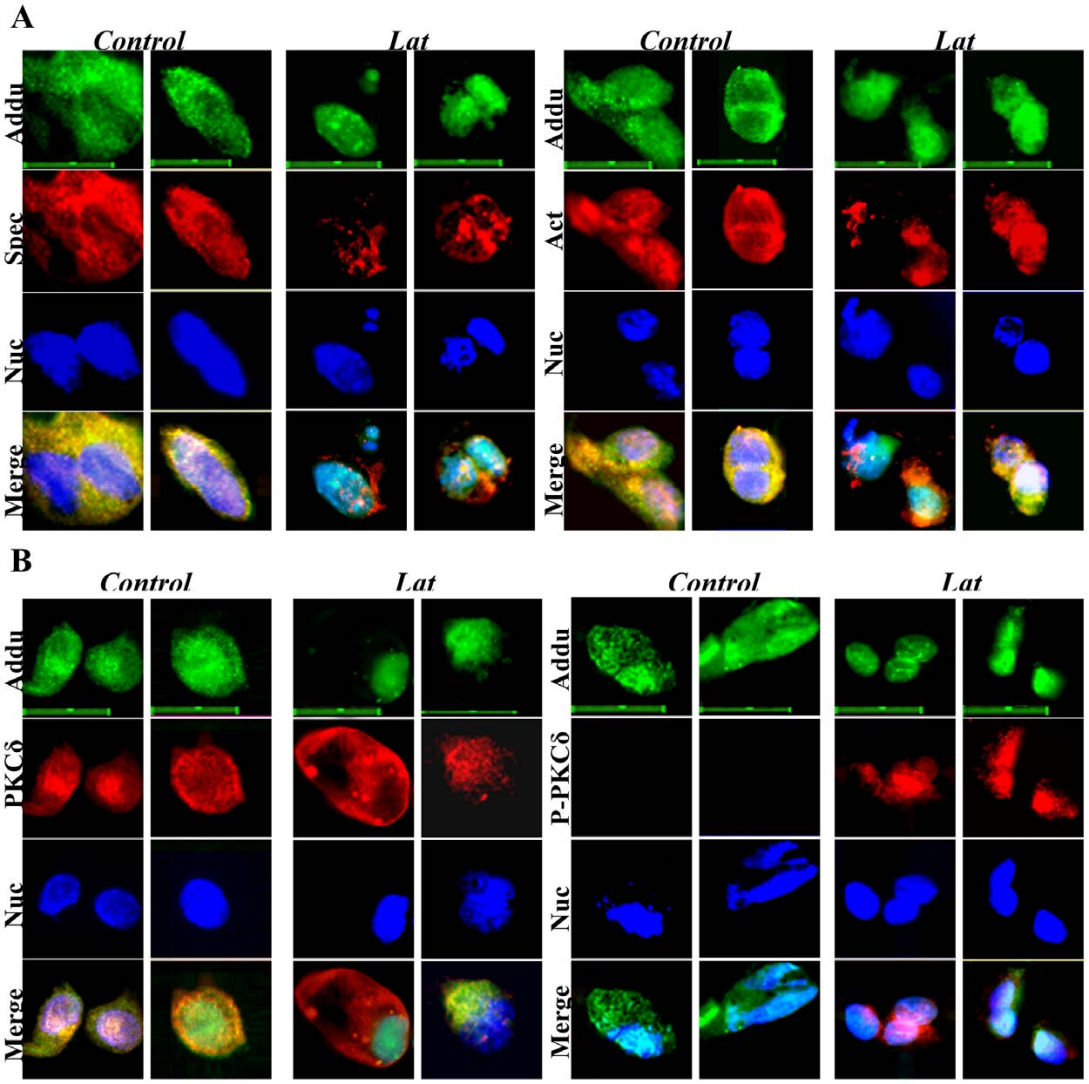
Co-expression of adducin with spectrin (A), actin (A), PKCδ (B) and P-PKCδ (B) in primary keratinocytes with or without Lat treatment. Control and Lat-treated keratinocytes were triple stained for adducin (Green), spectrin, actin, PKCδ and P-PKCδ (Red) and nuclei (Blue). Scale bars are 20 µm.

**Figure 8:**
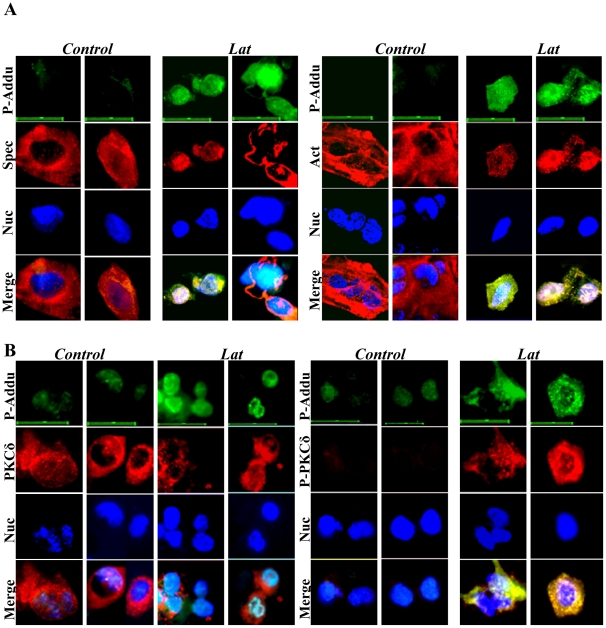
Co-expression of phosphorylated-adducin (P-Addu) with spectrin (A), actin (A), PKCδ (B) and P-PKCδ (B) in primary keratinocytes with or without Lat treatment. Control and Lat-treated keratinocytes were triple stained for P-Adducin (Green), spectrin, actin, PKCδ and P-PKCδ PKCδ (Red) and nuclei (Blue). Scale bars are 20 µm.

**Figure 9:**
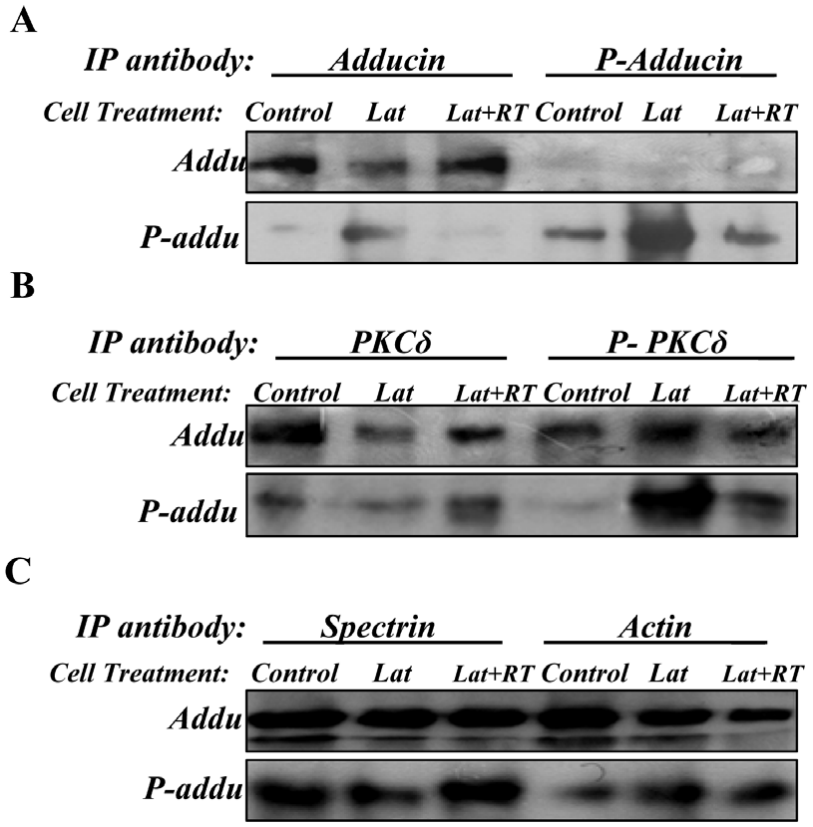
Immunoprecipitation assay of total proteins prepared from primary mouse keratinocyte cultures with the indicated antibodies. Primary mouse keratinocytes cultured for five days were treated with Latrunculin or with Latrunculin+Rottlerin for 12 h. Then the keratinocytes were collected for protein preparations. Protein samples were immunoprecipitated with six antibodies as indicated, respectively. (**A**). Proteins immunoprecipitated by antibodies against adducin and p-adducin were analyzed by immunoblotting using adducin and p-adducin antibodies as indicated. (**B**). Proteins immunoprecipitated with antibodies against PKCδ and p-PKCδ were analyzed by immunoblotting using adducin and p-adducin antibodies as indicated. (**C**). Proteins immunoprecipitated by antibodies against spectrin and actin were analyzed by immunoblotting using adducin and p-adducin antibodies as indicated. **Note:** IFNγ antibody was used as a control antibody for the IP experiments. As shown in Fig. 6, the Spectrin, PKCδ and P-PKCδ antibodies did not across react with the immunoprecipitates by IFNγ antibody. Neither adducin nor phosphorylated adducin antibody had a cross reaction with IFNγ antibody (data not shown).

Previous studies have reported that adducin, a cytoskeletal protein, is localized at spectrin–actin junctions to regulate assembly of spectrin-actin complexes in erythrocyte membrane skeletons [42] and PKC phosphorylates adducin and regulates its function [43]. Thus, we determined whether adducin and phosphorylated-adducin on Ser662 (P-adducin^(Ser662)^) were co-expressed with spectrin, actin, PKCδ and P-PKCδ in keratinocytes. Immunofluorescence staining showed that adducin is consistently co-expressed with spectrin, actin and PKCδ in differentiating keratinocytes (Fig. 7A, B). But, co-expression of adducin with P-PKCδ^(Thr505)^ was only observed in Lat-treated keratinocytes (Fig. 7B). Immunofluorescence staining showed co-expression of P-adducin with spectrin, actin, PKCδ and P-PKCδ^(Thr505)^ in Lat-treated keratinocytes (Fig. 8A, B). These data reveal that adducin is expressed in differentiating keratinocytes and suggest that P-adducin may be involved in re-organization of the spectrin-actin complex. To establish a role for adducin phosphorylation in this process, we investigated the effect of disruption of the spectrin-like cytoskeleton in the presence and absence of the PKCδ inhibitor RT. Under these conditions RT had a marked inhibitory effect on expression of P-adducin^(Ser662)^ (Fig. 9A). Immunoblotting analysis of immunoprecipitates showed further that both adducin and P-adducin^(Ser662)^ interacted with PKCδ, P-PKCδ^(Thr505)^, spectrin and actin (Fig. 9B,C). The strong interaction of adducin and P-adducin^(Ser662)^ with PKCδ and P-PKCδ^(Thr505)^ confirms that adducin is a potential substrate for PKCδ and P-PKCδ^(Thr505)^ in disruption of the spectrin-like cytoskeleton in keratinocytes.

**Figure 10:**
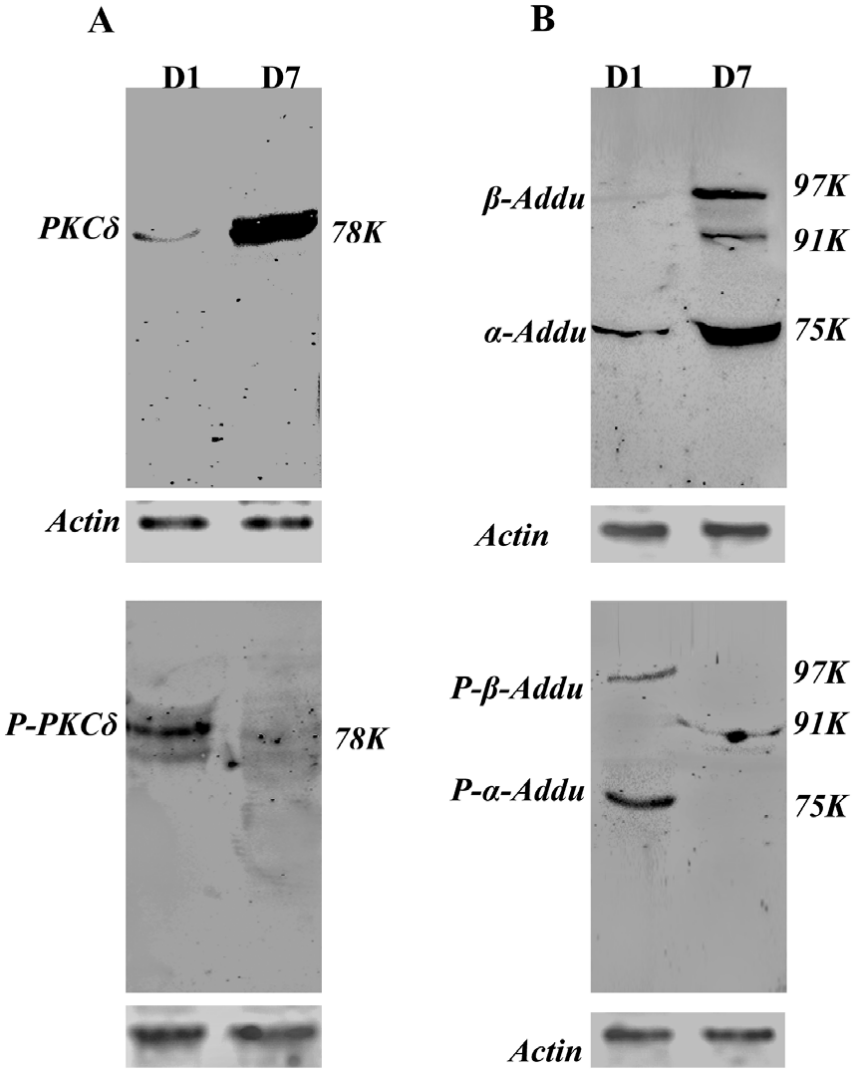
Western blot analysis for expression of (A) PKCδ and phosphorylated PKCδ^(Thr505)^ (P- PKCδ^(Thr505)^) and (B) adducin and phosphorylated adducin^(Ser662)^ (P-adducin^(Ser662)^) in primary mouse keratinocytes cultured for a period of one (D1) to seven (D7) days. Results are representative of two individual experiments.

We next examined whether and how PKCδ, P-PKCδ^(Thr505)^, adducin and p-adducin^(Ser662)^ were expressed in both D1 and D7 mouse primary keratinocyte cultures (Fig. 10A,B). Immunoblotting showed clearly that PKCδ was primarily expressed in D7 keratinocytes while P-PKCδ^(Thr505)^ was expressed in D1 keratinocytes (Fig. 10A). Immunoblotting showed further that adducin was largely expressed in D7 keratinocytes, with three bands corresponding to β-adducin at 94 kDa and α-adducin 74 kDa (Fig. 10B). Two bands of phosphorylated adducin were detected, corresponding to P-β-adducin^(Ser662)^ at 97 kDa and P-α-adducin^(Ser726)^ at 75 kDa in D1 keratinocytes (Fig. 10B). The data reveal two points: 1). both PKCδ and adducin have a similar expression pattern to that of spectrin in keratinocytes associated with cell differentiation and 2). expression of P-PKCδ^(Thr505)^ is coincident with that of P-β-adducin^(Ser662)^ in keratinocytes associated with cell proliferation.

### Knock-down of endogenous adducin disrupts spectrin-like cytoskeleton

**Figure 11:**
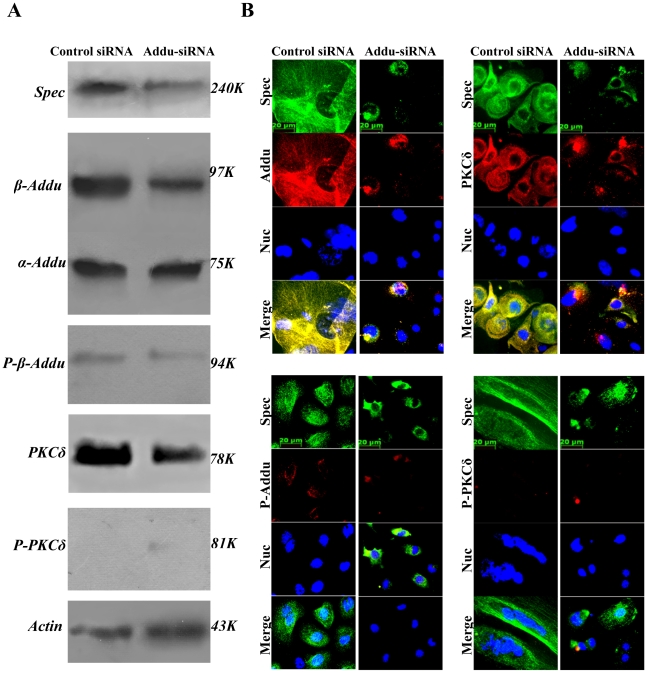
Effects of β-adducin specific siRNA on expression of spectrin, adducin, P-adducin^(Ser662)^, PKCδ and P-PKCδ^(Thr505)^ in primary mouse keratinocytes. Primary mouse keratinocytes, after culturing for five days, were transfected with either non-targeting siRNA (control siRNA) or β-adducin specific siRNA (Addu-siRNA), respectively. The siRNA-transfected keratinocytes at 48 h post-transfection were collected for both western blotting analysis and immunofluorescence labelling. (**A**). Western blot analysis for expression of spectrin, adducin, P-adducin^(Ser662)^, PKCδ and P-PKCδ^(Thr505)^. Actin was used as a loading control. Results are representative of two separate transfection experiments, with 6 replicates in one transfection experiment. (**B**). The keratinocytes transfected with control siRNA and adducin siRNA were triple-stained for spectrin (Green), P-adducin^(Ser662)^, adducin, PKCδ and P-PKCδ^(Thr505)^ (Red) and nuclei (Blue). Scale bars are shown in the images.

We determined whether knock-down of endogenous adducin in keratinocytes could affect expression of, spectrin, PKCδ and P-PKCδ^(Thr505)^ and lead to disruption of the spectrin-like cytoskeleton in D7 keratinocytes. We observed that β-adducin siRNAs-transfected keratinocytes had a distinctly different cell morphology from the control siRNA-transfected keratinocytes, with evidence of enhanced keratinocyte proliferation(Supporting information Fig. S10). Immunoblotting showed that transfection of the β-adducin siRNA resulted not only in significant reduction of adducin, but also of spectrin and PKCδ (Fig. 11A). The results confirm that expression of adducin was associated with that of spectrin and PKCδ in keratinocytes.

We next examined organization of the spectrin-like cytoskeleton compared with distribution of adducin, P-adducin^(Ser662)^, PKCδ and, P-PKCδ^(Thr505)^ in both control siRNA- and β-adducin siRNA-transfected keratinocytes by immunofluorescence staining. Control siRNA had no effects on organization of the spectrin-like cytoskeleton and the cytoskeletal organization as well as the distribution of both adducin and PKCδ in keratinocytes (Fig. 11B). In contrast, β-adducin siRNA transfection severely disrupted not only the organization of the spectrin-like cytoskeleton, but also the cytoskeletal organization and distribution of both PKCδ and adducin (Fig. 11B). The results provide evidence that organization of the spectrin-like cytoskeleton and spectrin expression are firmly dependent on either expression of endogenous PKCδ and adducin or phosphorylation of adducin activated by P-PKCδ^(Thr505)^ in differentiating keratinocytes.

## Discussion

Primary mouse and human epidermal keratinocytes grown *in vitro* can reproduce the general features of differentiation seen in suprabasal and granular cells of stratified epidermis (Fuchs and Green, 1980; Pillai et al, 1990). During keratinocyte differentiation and stratification of the epidermis, terminal differentiation markers including involucrin, transglutaminase and several keratins are expressed and assembled into intermediate filaments. Spectrin as a major plasma membrane-bond protein has been reported in the keratinocytes of human skin [17], [18], [26]. However, the studies showed different characteristics of the spectrin expressed in human skin. Different from the strong staining of the spectrin protein in differentiated keratinocytes in the granular and cornified layers of the human epidermis [17], Shimizu et al. observed that the spectrin proteins were only localized to the peripheral cytoplasm of the keratinocytes in the basal layer [26]. Tuominen et al. found that keratinocytes in all layers of the human skin expressed spectrin. While basal keratinocytes contained intracytoplasmic spectrin, suprabasal keratinocytes showed membrane-bound spectrin [18]. In fact, both cornifed and granular layers of human skin showed the strongest staining of the spectrin and well organized spectrin-like cytoskeleton. Here, we investigated expression of spectrin and its cytoskeletal organization in both mouse and human keratinocytes *in vitro* and *in vivo*. The results from the *in vitro* experiments clearly show that expression of spectrin protein and assembly of the spectrin-like cytoskeleton in primary keratinocytes cultures are tightly associated with keratinocyte differentiation, consistent with previous observations in erythrocytes [44]. Moreover, the observed changes of the spectrin-like cytoskeleton as a supportive membrane skeleton may reflect the membrane dynamics of the primary keratinocytes during cell differentiation. In epidermal keratinocytes, the membrane skeletal proteins play functional roles to maintain the polarity of membrane proteins by connecting them to the cytoskeleton, to regulate cell-cell interdigitations and stabilize newly synthesized cell membranes before elaboration of cell-cell interdigitations [26]. The spectrin-like cytoskeleton is thus an active material that can adapt its mechanics and perform structural tasks in mammalian epidermis. Furthermore, it is possible that the organized spectrin-like cytoskeleton can serve as an early end point for the development of skin.

Spectrin comprises α (240 kDa) and β (220 kDa) subunits, forming an elongated (αβ)2 tetramer in erythrocytes [1], but the full length proteins of the two subunits are not required to form the spectrin/adducin/actin complex [45]. Spectrin has been previously detected in keratinocytes [17], [18], [26], [46], [47], but it is unclear whether both α and β spectrins are expressed in full-length form to assemble into the spectrin-like cytoskeleton. In this study, we detected full-length α spectrin, but only a smaller size β spectrin at 78 kDa. The lower molecular size of β spectrin corresponds to an alternative N-terminal form (SPTBN4 with 678 amino acids; http://biogps.gnf.org). We have not established why only a short form of the β spectrin is expressed in differentiating keratinocytes, but our results are consistent with previous quantitative evidence showing that the β-spectrin N-terminal domain plus the first two α-helical domains are required for optimal participation of spectrin in spectrin/adducin/actin complexes [45].

We showed here that the spectrin-like cytoskeleton in the epidermis of mouse and human skin is prominently localized to the cornified and granular layers. The cornified envelope is a hallmark of terminal differentiation of keratinocytes, which is a highly insoluble and extremely tough structure formed beneath the cell membrane and is continuously replenished as granular keratinocytes move outwards and are sloughed from the skin surface [16]. The main function of the cornified envelope is to provide mammalian skin with a protective barrier that excludes harmful microbes and retains body fluids [14], [48]. The data presented here suggest that spectrin protein is a major structural component of granular keratinocytes and the cornified envelope. A previous report shows that non-erythrocyte spectrin is involved in linking intermediate filaments to the plasma membrane [49]. This suggests that spectrin may be a cross-linking substrate for interaction with multiple proteins to enhance the structural integrity of the epidermis and to generate the polarized morphology of epithelial cells [50], [51]. Thus, the spectrin-like cytoskeleton, prominently organized in the cornified envelope, may be involved in maintaining the cellular integrity and stratification of squamous epithelium in mouse and human skins and contribute to the barrier organization and function of the epidermis, which is fundamental to the physiology and development of epithelial cells [52].

Previous studies have shown that disassembly of a number of cytoskeletal structures including the nuclear lamina and vimentin containing intermediate filaments is induced during mitosis [53]. Tubulin phosphorylation is related to tubulin disassembly and architectural integrity of microtubules during the cell cycle [54]. According to the published studies in erythrocytes and other cells, spectrin phosphorylation occurs mainly on β spectrin [55], [56], [57]. Thus, in the present study, it is unlikely that the altered organization of the spectrin-like cytoskeleton observed in actin inhibitor-treated keratinocytes is due to phosphorylation of spectrin because no extra spectrin band was shown in inhibitor-treated keratinocytes. Furthermore, the reported phosphorylation of the cytoskeletal components and their functional changes are associated with cell cycle progression. CHO cell spectrin is phosphorylated exclusively on the β subunit in both interphase and mitotic cells [58]. Our results clearly indicate that spectrin expression and its cytoskeletal organization are tightly associated with keratinocyte differentiation and these cells exit the cell cycle once they enter the terminal differentiation program.

Treatment with the actin filament inhibitors (CB, STS and Lat) produced different effects on the organizations of microfilaments, spectrin-like cytoskeleton and PKCδ in keratinocytes. CB did not affect organizations of the spectrin-like cytoskeleton and PKCδ in keratinocytes although it disrupted microfilaments. Why CB does not disrupt organization of the spectrin-like cytoskeleton in keratinocytes can be explained by previous results [59], [60]. CB induced depolymerization of the endoplasmic reticulum (ER) -associated F-actin, but α-spectrin remained colocalized with the ER because it is bound to the ER membrane in Drosophila [59]. In the cytoskeletal ATPase fraction of human erythrocyte CB inhibited actin ATPase activity to polymerize G-actin to form shorter filaments, but did not affect spectrin polymerization because spectrin has the same elasticity as the underlying spectrin/dystrophin network and does not have the same cytoskeletal ATPase [60]. Both STS and Lat have been reported to be inhibitors of PKC, resulting in simultaneous redistribution and centralization of both spectrin and PKCδ and reduced expression of these proteins in differentiating keratinocytes [61], [62], [63]. Spectrin has been reported to colocalize with PKCβ II in T lymphocytes [64]. But, to date, no study has been reported showing that spectrin is co-expressed with any isoform of PKC in keratinocytes, although it has been reported that keratinocytes express two major isoforms of PKC (PKCα and PKCδ) [65]. Previous studies have well established that the novel PKC isoforms stimulate keratinocyte differentiation [66], [67]. PKCδ is the most potent of these activators [36], [68] and its activity can be inhibited by a specfic PKCδ inhibitor, rottlerin [65], [67], [69], we revealed here that PKCδ plays a regulatory role in organization of the spectrin-like cytoskeleton in keratinocytes.

Disruption of the spectrin-cytoskeleton by two microfilament inhibitors was also accompanied by markedly increased levels of PKCδ phosphorylation on Thr505. Previous data have shown that phosphorylation on this site increases the activity of this protein [37]. Thus it seems likely that PKCδ activation is causally linked to the re-organization of the spectrin-like cytoskeleton in keratinocytes. Under these conditions increased phosphorylation of PKCδ^(Thr505)^ was also observed. Further evidence for this was provided by inhibition of both PKCδ phosphorylation and disruption of the spectrin-like cytoskeleton by the PKCδ specific inhibitor rottlerin. This agent has previously been shown to be anti-proliferative, inhibiting cell cycle progression and enhancing apoptosis [70]. Since there is no evidence that PKCδ phosphorylates spectrin or actin it seemed likely that the disassembly of spectrin-actin complexes would be mediated through an intermediate substrate. A good candidate for this role is adducin, a protein that preferentially recruits spectrin to the ends of growing actin filaments to stabilize the complex [71], [72]. Adducin is localized at spectrin–actin junctions in erythrocyte membrane skeletons and colocalizes with spectrin at sites of cell–cell contact in epithelial cells [46], [71], [73]. We have shown here that adducin is involved in the formation of a spectrin-actin lattice in keratinocytes. Previous results have only reported that it is present in the epidermis [46]. PKC phosphorylation of native or recombinant adducin inhibited F-actin capping and prevented the recruitment of spectrin to actin filaments [72]. They also demonstrated that PKC phosphorylated adducin in neuronal dendritic spines that respond to external stimuli by changing the morphology and re-organization of cytoskeletal structures. But it was unclear whether a specific form of PKC was phosphorylated. Phosphorylation of adducin also occurs during platelet activation causing its dissociation from the spectrin-actin complex which in turn leads to release of spectrin from actin [74]. This leads to exposure of actin filaments which may contribute to the platelet shape change on activation. We have demonstrated here disruption of the spectrin-like cytoskeleton by one of the actin filament inhibitors latrunculin. Furthermore, the PKCδ inhibitor rottlerin prevented latrunculin-induced phosphorylation of adducin and the accompanying disruption of the spectrin-like cytoskeleton. Previous reports on the phosphorylation of adducin in hippocampal neurons, platelets, growth cones of axons and in erythrocytes provide evidence for an involvement of PKC but none of these identified the specific PKC subtype involved [71], [73], [74], [75], [76]. Our data reveal that PKCδ contributes to this process. However, it is likely that this signaling is complex involving more than one phosphorylation site on adducin, more than one protein kinase and participation of substrates in addition to adducin. We showed here that adducin is phosphorylated on ser662 but two other PKC-dependent phosphorylation events (ser716 and ser726) on adducin inhibit its activity in promoting spectrin-actin complex formation [72]. While the outcome may be the same in the different systems, disruption of the spectrin-actin complex, this appears to be achieved by adducin phosphorylation at different sites in response to different stimuli. Rho-kinase has also been shown to phosphorylate adducin on Thr445 which appears to have the opposite effect to PKC-dependent phosphorylation in that it positively regulates the association of adducin with the spectrin-actin cytoskeleton [77]. A variety of other proteins including protein 4.1R, band 3/ankyrin complex, tropimodulin and tropomyosin are also involved in the assembly. Furthermore, knock-down of endogenous adducin by β-adducin siRNA disrupted the spectrin-like cytoskeleton and reduced expression of spectrin in keratinocytes. Although the adducin-β siRNA significantly reduced expression of both adducin and PKCδ and disrupted their cytoskeletal distributions, it did not activate adducin on Ser662 and PKCδ on Thr505 in keratinocytes. Thus, it appears that adducin plays different roles in mediating the organization of the spectrin-like cytoskeleton in keratinocytes. The phosphorylation event may not be the only means of activating PKCδ since Zhu et al (2008) have shown that tyrosine phosphorylation on Y311 as well as on other secondary sites influence the ability of PKCδ to increase expression of involucrin. Nevertheless, the organization of the spectrin-like cytoskeleton and spectrin expression in differentiating keratinocytes are tightly associated with adducin expression and its physiological status. Adducin acts through spectrin to stabilize epithelial junctions and to regulate global properties of the membrane of keratinocytes.

In summary, we show for the first time that the spectrin-like cytoskeleton assembled in mouse and human keratinocytes is dynamically associated with cell differentiation. The spectrin-like cytoskeleton organized in differentiating keratinocytes and its predominantly regional localization to the cornified layer of the epidermis is essential for healthy human skin. The spectrin-like cytoskeleton interacts with actin filaments to form a ternary complex to support the keratinocyte plasma membrane and the integrity of the complex is dependent on PKCδ activity, adducin phosphorylation status and cell differentiation. Both activation of PKCδ (Thr505) by microfilament inhibitors and knock-down of endogenous adducin by adducin specific siRNA remodel the spectrin-actin ternary complex. The results have important implications for the spectrin-like cytoskeleton and its potential as a model system for studying the cellular integrity and stratification of keratinocytes during differentiation, the mechanisms that regulate skin development and the barrier organization and function of the epidermis.

### Supplementary Material

Total 10 supplementary figures include results on spectrin expression and cytoskeleton in human primary keratinocytes, staining of spectrin, actin, tubulin, K14 and involucrin in both mouse and human skin sections and the results from different experiments.

## Supporting Information

Figure S1 Organization of spectrin-like cytoskeleton in primary human keratinocytes *in vitro* cultured for seven days. Cells were compared for organization of spectrin-like and microtubule cytoskeleton in primary keratinocyte cultures for **D1** and **D7** by immunofluorescence staining. Scale bars are 20 µm.
(DOC)

Figure S2 Expression of spectrin in primary mouse and human keratinocytes *in vitro* cultured for seven days. (**A**). Western blotting analysis of spectrin, involucrin and actin in primary mouse keratinocyte cultures for **D1**, **D4** and **D7**. (**B**). Western blotting analysis of spectrin, involucrin and tubulin in primary human keratinocytes cultured for **D1**, **D4** and **D7**. Forty micrograms of protein samples were loaded for Western blotting analysis.
(DOC)

Figure S3 Spectrin-like and tubulin cytoskeletons in mouse and human skin. Skin sections were immunostained as indicated for spectrin (Green) and tubulin (Red). Nuclei (Blue) from the same fields were counterstained with DAPI. Epidermis (E), dermis (D) and hair follicles (H) of the skin sections are indicated.
(DOC)

Figure S4 Spectrin-like cytoskeleton and K14 filament in mouse and human skins. Skin sections were immunostained as indicated for spectrin (Green) and K14 (Red). Nuclei (Blue) from the same fields were counterstained with DAPI.
(DOC)

Figure S5 Spectrin-like cytoskeleton and involucrin filament in mouse and human skin. Skin sections were immunostained as indicated for spectrin (Green) and involucrin (Red). Nuclei (Blue) from the same fields were counterstained with DAPI.
(DOC)

Figure S6 Disruption of actin filaments in primary mouse keratinocytes. Primary mouse keratinocytes after culturing for five days were treated with microtubule and microfilament inhibitors for 12 h, respectively. Both control and treated keratinocytes were triple stained for actin (green), tubulin (red) and nuclei (blue).
(DOC)

Figure S7 Effects of two microfilament inhibitors straurosporine (STS) and latrunculin B [21] on spectrin expression in primary mouse keratinocyte cultures. Primary mouse keratinocytes after culturing for five days were treated with three inhibitors for 12 h, respectively. Western blot analysis showed expression of spectrin, with the major band at 240 kDa and another band approximately at 120 kDa. The two inhibitors did not produce additional bands in keratinocytes except for reduced expression of the spectrin.
(DOC)

Figure S8 Microfilament inhibitors (CB, STS and Lat) had no effects on expression of PKCα in primary mouse keratinocytes. Primary mouse keratinocytes after culturing for five days were treated with three inhibitors for 12 h, respectively. Western blot analysis for expression of PKCα, tubulin and actin.
(DOC)

Figure S9 Immunoprecipitation assay of total proteins prepared from primary mouse keratinocyte cultures with the indicated antibodies. Primary mouse keratinocytes cultured for five days were treated with or without Latrunculin for 12 h. The keratinocytes were then collected for protein preparations. Proteins were immunoprecipitated with four antibodies as indicated, respectively, and analyzed by immunoblotting assay using five antibodies, respectively.
(DOC)

Figure S10 Morphology of control- and adducin β-siRNA transfected mouse primary keratinocytes at 44 h post-transfection. A, B, C and D representatives of four wells of the siRNA transfected keratinocytes. Images were taken using 10× objective lens.
(DOC)
