# Supplementary material for: Disruption of Spectrin-Like Cytoskeleton in Differentiating Keratinocytes by PKCδ Activation Is Associated with Phosphorylated Adducin
Source: PLoS One. 2011 Dec 7;6(12):e28267. doi: 10.1371/journal.pone.0028267 (PMC3233558; doi:10.1371/journal.pone.0028267)
Supplement: Figure S1 — Organization of spectrin-like cytoskeleton in primary human keratinocytes in vitro cultured for seven days. Cells were compared for organization of spectrin-like and microtubule cytoskeleton in primary keratinocyte cultures for D1 and D7 by immunofluorescence staining. Scale bars are 20 µm. (DOC) [file pone.0028267.s001.doc]

**Supplementary data:**

**Supporting information Fig. S1**

**D1 D7**

**
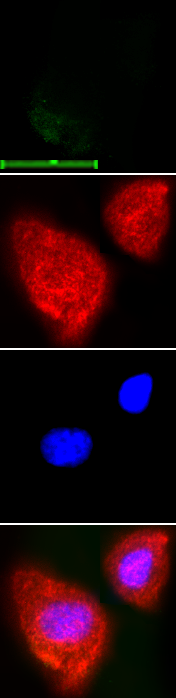

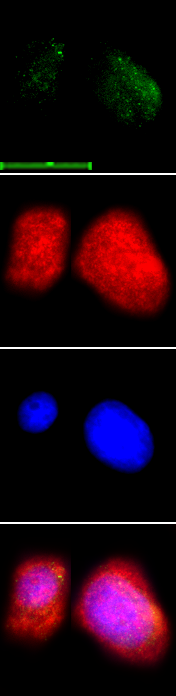

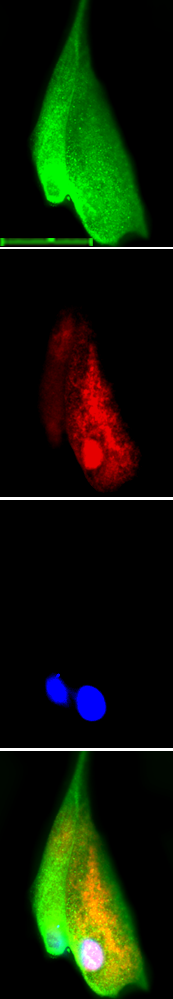

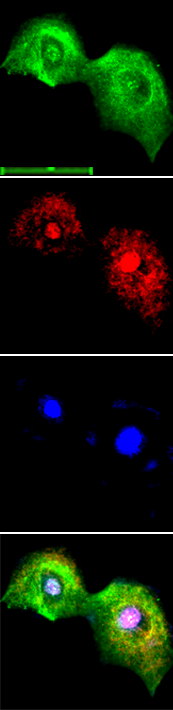
**

**Merge Nuc Tub Spec**

**Fig. S1.** Organization of spectrin-like cytoskeleton in primary human keratinocytes in vitro cultured for seven days. Cells were compared for organization of spectrin-like and microtubule cytoskeleton in primary keratinocyte cultures for **D1** and **D7** by immunofluorescence staining. Scale bars are 20 µm.
