# Supplementary material for: Disruption of Spectrin-Like Cytoskeleton in Differentiating Keratinocytes by PKCδ Activation Is Associated with Phosphorylated Adducin
Source: PLoS One. 2011 Dec 7;6(12):e28267. doi: 10.1371/journal.pone.0028267 (PMC3233558; doi:10.1371/journal.pone.0028267)
Supplement: Figure S2 — Expression of spectrin in primary mouse and human keratinocytes in vitro cultured for seven days. (A). Western blotting analysis of spectrin, involucrin and actin in primary mouse keratinocyte cultures for D1, D4 and D7. (B). Western blotting analysis of spectrin, involucrin and tubulin in primary human keratinocytes cultured for D1, D4 and D7. Forty micrograms of protein samples were loaded for Western blotting analysis. (DOC) [file pone.0028267.s002.doc]

**Supporting information Fig. S2**

**B.**


**D1 D4 D7**

**D1 D4 D7**

**
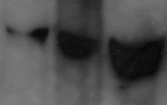
**


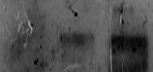

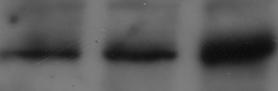

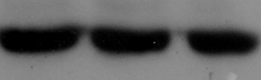


**Tub Invol Spec**

**Act Invol Spec**

**
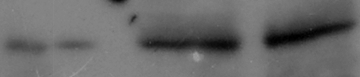
**

**
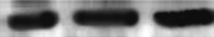
**

**Fig. S2.** Expression of spectrin in primary mouse and human keratinocytes in vitro cultured for seven days. **(A)**. Western blotting analysis of spectrin, involucrin and actin in primary mouse keratinocyte cultures for **D1**, **D4** and **D7.**  **(B).** Western blotting analysis of spectrin, involucrin and tubulin in primary human keratinocytes cultured for **D1**, **D4** and **D7**. Forty micrograms of protein samples were loaded for Western blotting analysis.
