# Supplementary material for: Disruption of Spectrin-Like Cytoskeleton in Differentiating Keratinocytes by PKCδ Activation Is Associated with Phosphorylated Adducin
Source: PLoS One. 2011 Dec 7;6(12):e28267. doi: 10.1371/journal.pone.0028267 (PMC3233558; doi:10.1371/journal.pone.0028267)
Supplement: Figure S3 — Spectrin-like and tubulin cytoskeletons in mouse and human skin. Skin sections were immunostained as indicated for spectrin (Green) and tubulin (Red). Nuclei (Blue) from the same fields were counterstained with DAPI. Epidermis (E), dermis (D) and hair follicles (H) of the skin sections are indicated. (DOC) [file pone.0028267.s003.doc]

**Supporting information Fig. S3**

**Mouse Human**

**
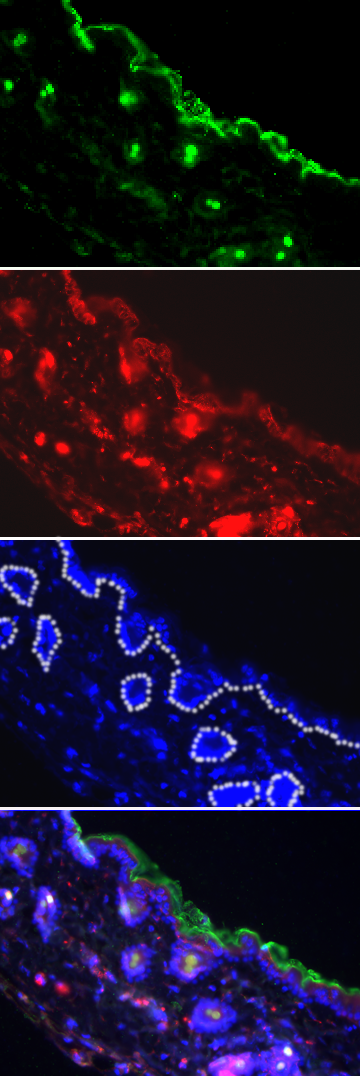

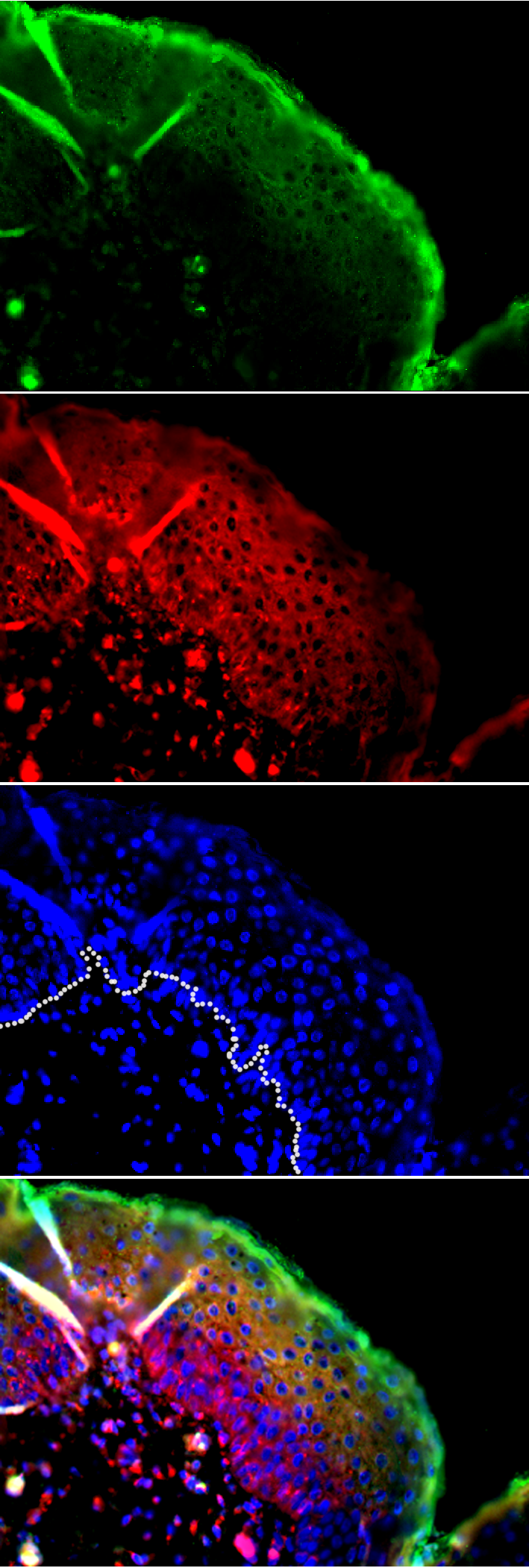
**

**Spectrin**

**Tubulin**

**Nucleus**

**Merge**

**ii**

**E**

**H**

**H**

**H**

**E**

**E**

**E**

**H**

**H**

**H**

**H**

**H**

**D**

**D**

**D**

**D**

**D**

**E**

**E**

**E**

**D**

**D**

**E**

**E**

**Fig. S3.** Spectrin-like and tubulin cytoskeletons in mouse and human skin. Skin sections were immunostained as indicated for spectrin (Green) and tubulin (Red). Nuclei (Blue) from the same fields were counterstained with DAPI. Epidermis (E), dermis (D) and hair follicles (H) of the skin sections are indicated.
