# Supplementary material for: Disruption of Spectrin-Like Cytoskeleton in Differentiating Keratinocytes by PKCδ Activation Is Associated with Phosphorylated Adducin
Source: PLoS One. 2011 Dec 7;6(12):e28267. doi: 10.1371/journal.pone.0028267 (PMC3233558; doi:10.1371/journal.pone.0028267)
Supplement: Figure S4 — Spectrin-like cytoskeleton and K14 filament in mouse and human skins. Skin sections were immunostained as indicated for spectrin (Green) and K14 (Red). Nuclei (Blue) from the same fields were counterstained with DAPI. (DOC) [file pone.0028267.s004.doc]

**Supporting information Fig. S4**

**Mouse Human**


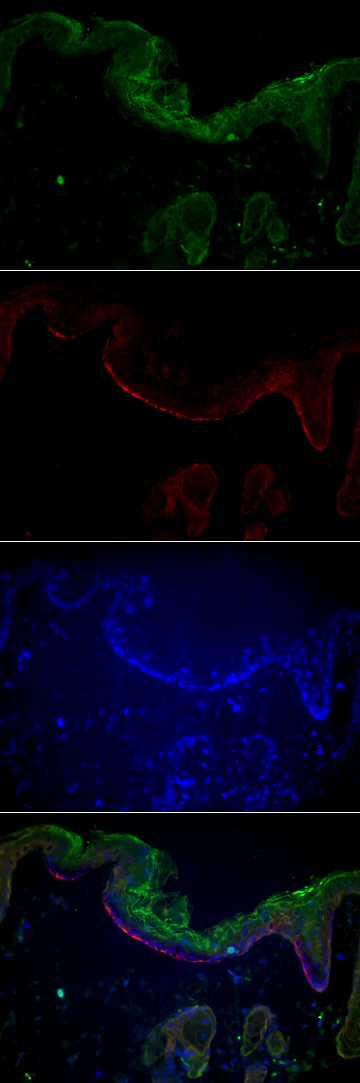

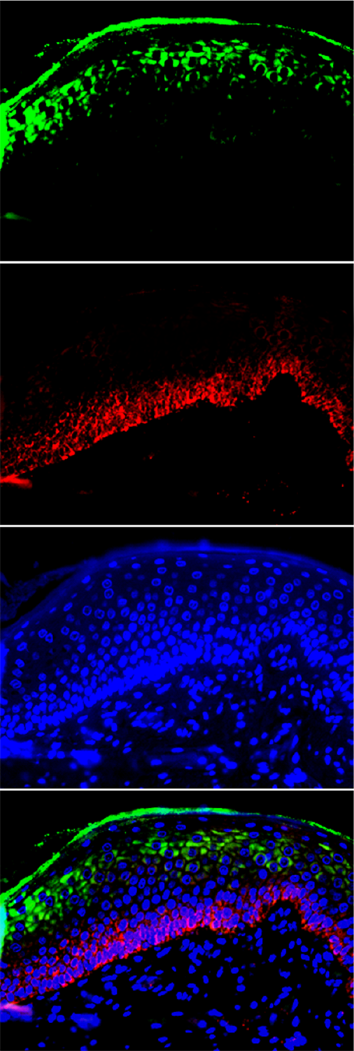


**Merge Nucleus K14 Spectrin**

**Fig. S4.** Spectrin-like cytoskeleton and K14 filament in mouse and human skins. Skin sections were immunostained as indicated for spectrin (Green) and K14 (Red). Nuclei (Blue) from the same fields were counterstained with DAPI.
