# Supplementary material for: Disruption of Spectrin-Like Cytoskeleton in Differentiating Keratinocytes by PKCδ Activation Is Associated with Phosphorylated Adducin
Source: PLoS One. 2011 Dec 7;6(12):e28267. doi: 10.1371/journal.pone.0028267 (PMC3233558; doi:10.1371/journal.pone.0028267)
Supplement: Figure S5 — Spectrin-like cytoskeleton and involucrin filament in mouse and human skin. Skin sections were immunostained as indicated for spectrin (Green) and involucrin (Red). Nuclei (Blue) from the same fields were counterstained with DAPI. (DOC) [file pone.0028267.s005.doc]

**Supporting information Fig. S5**

**Mouse Human**


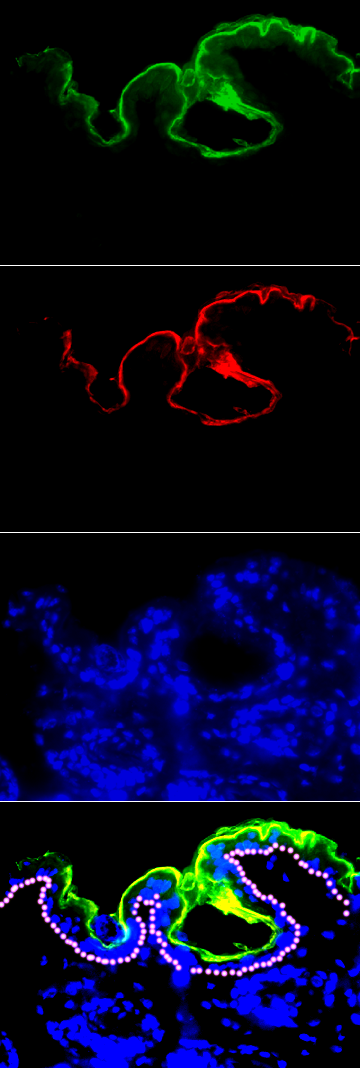

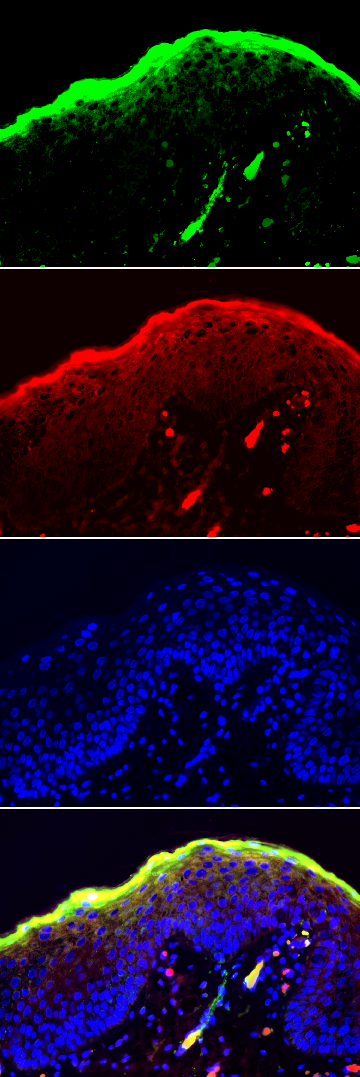


**Merge Nucleus Involucrin Spectrin**

**Spectrin**

**Fig. S5.** Spectrin-like cytoskeleton and involucrin filament in mouse and human skin. Skin sections were immunostained as indicated for spectrin (Green) and involucrin (Red). Nuclei (Blue) from the same fields were counterstained with DAPI.
