# Supplementary material for: Disruption of Spectrin-Like Cytoskeleton in Differentiating Keratinocytes by PKCδ Activation Is Associated with Phosphorylated Adducin
Source: PLoS One. 2011 Dec 7;6(12):e28267. doi: 10.1371/journal.pone.0028267 (PMC3233558; doi:10.1371/journal.pone.0028267)
Supplement: Figure S6 — Disruption of actin filaments in primary mouse keratinocytes. Primary mouse keratinocytes after culturing for five days were treated with microtubule and microfilament inhibitors for 12 h, respectively. Both control and treated keratinocytes were triple stained for actin (green), tubulin (red) and nuclei (blue). (DOC) [file pone.0028267.s006.doc]

**Supporting information Fig. S6**

***Conl Col Noc***


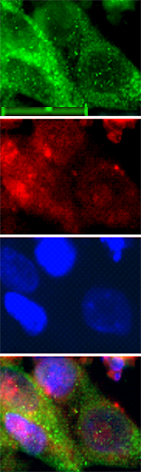

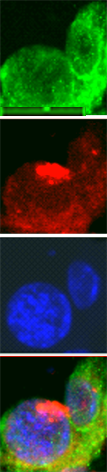

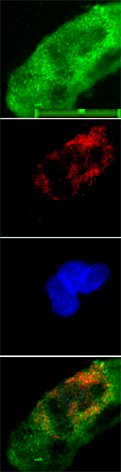

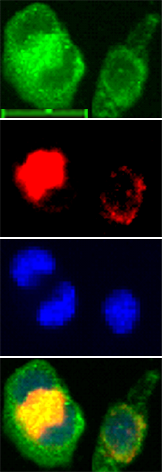

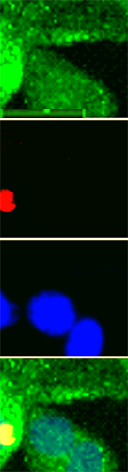

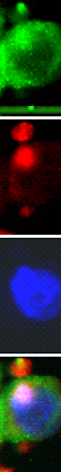


**Merge Nucl Tub Act**

***CB STS Lat***


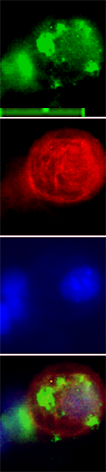

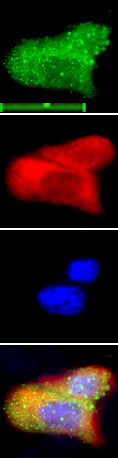

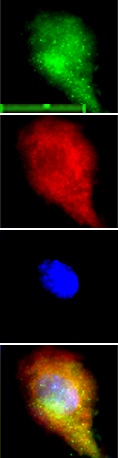

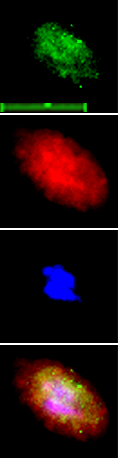

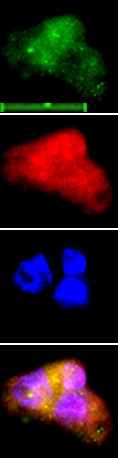

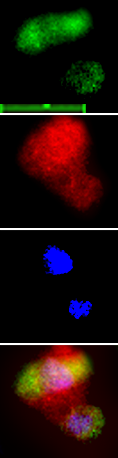


**Merge Nucl Tub Act**

**Fig. S6.** Disruption of actin filaments in primary mouse keratinocytes. Primary mouse keratinocytes after cultured for five days were treated with microtubule and microfilament inhibitors for 12 hr, respectively. Both control and treated keratinocytes were triple stained for actin (green), tubulin (red) and nucleus (blue).
