# Supplementary material for: Disruption of Spectrin-Like Cytoskeleton in Differentiating Keratinocytes by PKCδ Activation Is Associated with Phosphorylated Adducin
Source: PLoS One. 2011 Dec 7;6(12):e28267. doi: 10.1371/journal.pone.0028267 (PMC3233558; doi:10.1371/journal.pone.0028267)
Supplement: Figure S7 — Effects of two microfilament inhibitors straurosporine (STS) and latrunculin B [21] on spectrin expression in primary mouse keratinocyte cultures. Primary mouse keratinocytes after culturing for five days were treated with three inhibitors for 12 h, respectively. Western blot analysis showed expression of spectrin, with the major band at 240 kDa and another band approximately at 120 kDa. The two inhibitors did not produce additional bands in keratinocytes except for reduced expression of the spectrin. (DOC) [file pone.0028267.s007.doc]

**Supporting information Fig. S7**

***kDa Mark Conl STS Lat***

**
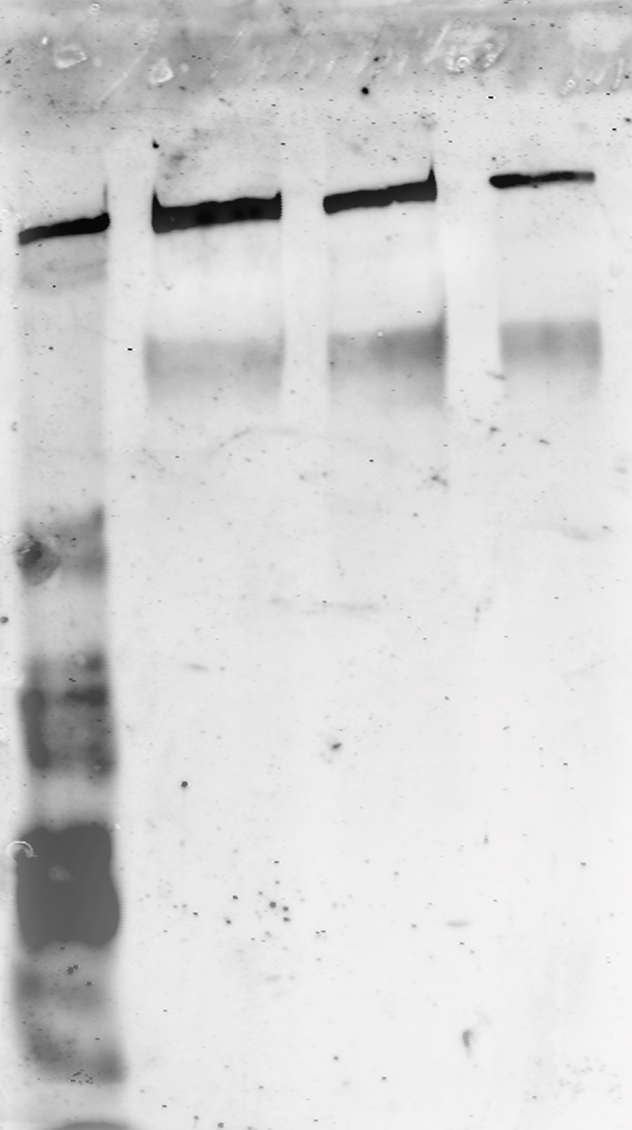
**

**250**

**148**

**98**

**64**

**50**

**36**

**22**

**16**

**6**

**4**

**240 kDa**

**120 kDa**

**Fig. S7.** Effects of two microfilament inhibitors straurosporine (STS) and latrunculin B (Lat) on spectrin expression in primary mouse keratinocyte cultures. Primary mouse keratinocytes after culturing for five days were treated with three inhibitors for 12 h, respectively. Western blot analysis showed expression of spectrin, with the major band at 240 kDa and another band approximately at 120 kDa observed. The two inhibitors did not produce additional bands in keratinocytes except for reduced expression of the spectrin.
