# Supplementary material for: Disruption of Spectrin-Like Cytoskeleton in Differentiating Keratinocytes by PKCδ Activation Is Associated with Phosphorylated Adducin
Source: PLoS One. 2011 Dec 7;6(12):e28267. doi: 10.1371/journal.pone.0028267 (PMC3233558; doi:10.1371/journal.pone.0028267)
Supplement: Figure S8 — Microfilament inhibitors (CB, STS and Lat) had no effects on expression of PKCα in primary mouse keratinocytes. Primary mouse keratinocytes after culturing for five days were treated with three inhibitors for 12 h, respectively. Western blot analysis for expression of PKCα, tubulin and actin. (DOC) [file pone.0028267.s008.doc]

**Supporting information Fig. S8**


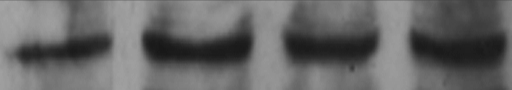

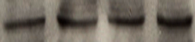


***Conl CB STS Lat***

***PKCα***

***Tub***

***Act***


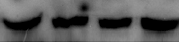


**Fig S8.** Microfilament inhibitors (CB; STS and Lat) had no effects on expression of PKCα in primary mouse keratinocytes. Primary mouse keratinocytes after culturing for five days were treated with three inhibitors for 12 h, respectively. Western blot analysis for expression of PKCα, tubulin and actin.
