# Supplementary material for: Disruption of Spectrin-Like Cytoskeleton in Differentiating Keratinocytes by PKCδ Activation Is Associated with Phosphorylated Adducin
Source: PLoS One. 2011 Dec 7;6(12):e28267. doi: 10.1371/journal.pone.0028267 (PMC3233558; doi:10.1371/journal.pone.0028267)
Supplement: Figure S9 — Immunoprecipitation assay of total proteins prepared from primary mouse keratinocyte cultures with the indicated antibodies. Primary mouse keratinocytes cultured for five days were treated with or without Latrunculin for 12 h. The keratinocytes were then collected for protein preparations. Proteins were immunoprecipitated with four antibodies as indicated, respectively, and analyzed by immunoblotting assay using five antibodies, respectively. (DOC) [file pone.0028267.s009.doc]

**Supporting information Fig. S9**

***IP Antibody: Invol Tub Pan-PKC PKCα***


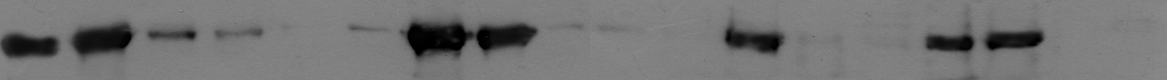

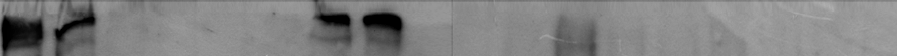

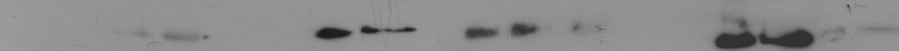

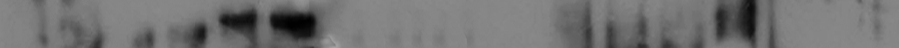

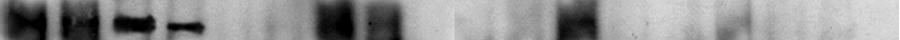


***Treatment: Conl Lat Conl Lat Conl Lat Conl Lat***

***Spec***

***PKCδ***

***P-PKCδ***

***Act***

***IF-γ***

**Fig. S9.** Immunoprecipitation assay of total proteins prepared from primary mouse keratinocyte cultures with the indicated antibodies. Primary mouse keratinocytes cultured for five days were treated with or without Latrunculin for 12 h. The keratinocytes were then collected for protein preparations. Proteins were immunoprecipitated with four antibodies as indicated, respectively, and analyzed by immunoblotting assay using five antibodies, respectively.
