# Supplementary material for: Disruption of Spectrin-Like Cytoskeleton in Differentiating Keratinocytes by PKCδ Activation Is Associated with Phosphorylated Adducin
Source: PLoS One. 2011 Dec 7;6(12):e28267. doi: 10.1371/journal.pone.0028267 (PMC3233558; doi:10.1371/journal.pone.0028267)
Supplement: Figure S10 — Morphology of control- and adducin β-siRNA transfected mouse primary keratinocytes at 44 h post-transfection. A, B, C and D representatives of four wells of the siRNA transfected keratinocytes. Images were taken using 10× objective lens. (DOC) [file pone.0028267.s010.doc]

**Supporting information Fig. S10**


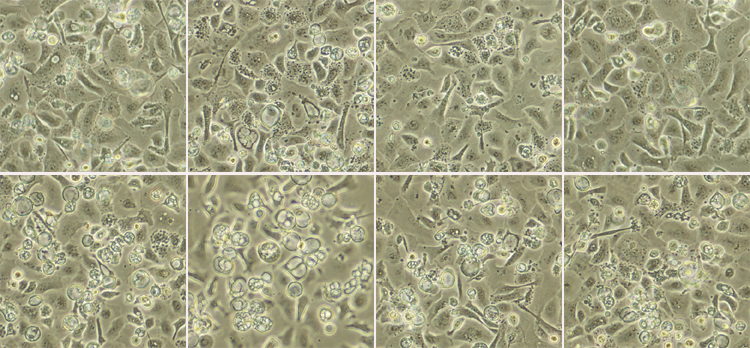


**Adducin β-siRNA Control siRNA**

**A B C D**

**Fig. S10.** Morphology of control- and adducin β- siRNA transfected mouse primary keratinocytes at 44 h post transfection. A, B, C and D representatives of four wells of the siRNA transfected keratinocytes. (Images taken using 10x objective lens)
